# Great Challenges Remain for niPGT-A Reliability

**DOI:** 10.1055/s-0042-1744291

**Published:** 2022-06-07

**Authors:** Bruno Ramalho de Carvalho

**Affiliations:** 1 Bruno Ramalho Reprodução Humana, Brasília, DF, Brazil

In the case report *First Baby Born in Brazil after Simultaneous Diagnosis through Non-Invasive and Conventional PGT-A* (Rev Bras Ginecol Obstet. 2021;43[11]), Kulmann et al. 1 present noninvasive preimplantation genetic test for aneuploidies (niPGT-A) as an alternative to conventional PGT-A. Those who defend the new technology assume that the biopsy of the trophectoderm could affect embryo health and its implantation potential. Also, the proposed technique assumes that the cell-free DNA found in the spent culture media (SCM) represents the genetic status of the embryo. However, as highlighted in their *Introduction* , the concordance rates between trophectoderm and SCM samples have been reported to greatly vary among studies, from insufficient ∼ 30% (Vera-Rodriguez et al., 2018) 2 to amazing ∼ 94% (Huang et al., 2019). 3 A critical look at this discrepancy leads the observer to realize that a lot of progress needs to be made before introducing that technique into the routine of the reproductive clinic.

Two studies by Rubio et al. (2019, 2020) 4 5 seem to be the pillars of this case report. The second study (Rubio et al., 2020) 5 is really interesting, since it included 1,301 human blastocysts, with promising concordance rates demonstrated. But uncertainties on the need to extend embryo culture to days 6 and 7, and the theoretical loss on reproductive potential of such blastocysts compared to day-5 ones are still concerning. Even with low-quality evidence, some studies suggest better clinical pregnancy and live birth rates in favor of day 5 blastocysts. 6 7 8 9 10 11

Considering the expectation of presenting an accurate test, it seems intriguing to find out that total concordance between conventional and niPGT-A occurred only for 3/7 blastocysts in the presented case. In real life, partial concordance is a non-encouraging result, and I would dare to say that it is as inutile as a total discordance. Of note, other recent studies present frustrating concordance rates for autosomes or sex chromosomes between trophectoderm or inner cell mass, and SCM analyses. 12 13

Finally, it is important to highlight the optimization of culture conditions, SCM retrieval, DNA isolation, and amplification protocols as great challenges for niPGT-A reliability. To date, we must be concerned about the theoretical high risk of maternal contamination. 14 15 Fortunately, the authors indicate that niPGT-A is not ready to replace conventional technique in routine and that further studies are needed to lead science in the best direction.
